# Experimental investigation of droplet impact behavior considering leaf curvature and vibration effects

**DOI:** 10.1186/s12870-025-08027-y

**Published:** 2025-12-30

**Authors:** Zhouming Gao, Jinlong Lin, Jing Ma, Wei Hu, Xiaoya Dong, Baijing Qiu

**Affiliations:** 1 School of engineering, Jiangxi Agricultural University, Nanchang, 330045 China; 2 School of agricultural engineering, Jiangsu University, Zhenjiang, Jiangsu 212013 China; 3 School of Mechanical Engineering, Zhejiang University of Water Resources and Electric Power, Hangzhou, 310018 China

**Keywords:** Droplet, Leaf curvature, Vibration, High-speed imaging, Impact behavior, Pesticide spraying

## Abstract

**Background:**

The impact behavior of droplets on crop leaves is a key factor in evaluating pesticide spray effectiveness. However, the coupled influences of the Weber number (*We*), leaf curvature (C^*^), and leaf vibration frequency (*f*) on droplet impact dynamics remain insufficiently understood.

**Results:**

By independently regulating *We*, C^*^, and *f* and using high-speed imaging, we found that higher leaf curvature caused asymmetric spreading, with the maximum diameter increasing by 6.89% along the *x*-axis and decreasing by 1.95% along the *y*-axis. At high *We* (≥ 168), spreading duration was reduced by at least 35.88%, while splashing probability increased. Vibration experiments showed that droplet-leaf motion shifted from synchronous (*θ*_*p*_ → 0) to counter-rotating (*θ*_*p*_ → *π*) as *f* increased from 10 to 80 Hz. Within the resonance range (40–50 Hz), both spreading and amplitude reached peak values, accompanied by the highest splashing risk. A quadratic regression model developed from a three-factor orthogonal design identified *We* and *f* as the dominant factors influencing maximum spreading (*P* < 0.05; *We* > *f* > C^*^).

**Conclusion:**

This study clarifies the coupled roles of *We*, C^*^, and *f* in droplet-leaf interactions and suggests maintaining *We* < 132 in practical spraying. Under typical conditions, droplet impact velocity should be kept at 3–5 m/s, and reduced to 2–3 m/s for larger droplets (> 500 μm). To avoid resonance-induced splashing, airflow in air-assisted spraying should be controlled at 6–10 m/s. These findings provide guidance for improving pesticide deposition and optimizing spray practices.

## Background

Droplet impingement on solid surfaces occurs in both natural and industrial processes and has been widely investigated in multiple disciplines, such as crop-protection spraying [1] and surface coating [2]. In crop-protection applications, droplet impact on leaf surfaces is critical because it determines spray deposition efficiency [3]. In recent years, substantial progress has been made in understanding droplet impact behavior on static leaves or rigid surfaces. However, under actual field conditions, plant leaves exhibit curvature and are subject to vibrations induced by external disturbances such as wind or impinging droplets. This coupled “curvature-vibration” state remains insufficiently explored, hindering a comprehensive understanding of droplet dynamics under realistic spraying scenarios and constraining the precise optimization of air-assisted spray parameters.

The impact behavior of droplets is jointly governed by the intrinsic properties of the droplets and the surface characteristics of crop leaves. Previous studies have systematically elucidated the fundamental roles of droplet properties such as surface tension, impact velocity, and droplet size in shaping impact dynamics [4]– [5]. For example, by adjusting spray formulations to modify droplet surface tension and employing high-speed imaging, researchers have clearly observed differences in adhesion, rebound, and fragmentation on leaves with varying wettability and surface roughness, thereby revealing the interaction mechanisms between droplet attributes and leaf surface features [6]– [7]. In addition, experimental studies combined with numerical simulations have demonstrated that droplet size and impact velocity not only determine inertial forces during impact but also jointly influence the maximum spreading diameter, and established trends show that spreading first increases and then decreases with velocity while increasing monotonically with droplet size [8]. Furthermore, Delele et al. [9] and An et al. [10] integrated parameters such as droplet size and impact velocity into a unified analytical framework using dimensionless numbers such as the Weber number (*We*). Their work systematically revealed how droplet-related dimensionless parameters regulate the maximum spreading factor and established predictive models and transition thresholds for spreading, rebound, and fragmentation behaviors.

In addition to the intrinsic properties of droplets, the surface characteristics of crop leaves also play a crucial role in governing droplet impact dynamics. Existing studies have shown that leaf roughness and wettability, as key material properties, can influence interfacial energy dissipation and contact-line motion, thereby affecting droplet adhesion, spreading, and retraction behaviors [6]– [7]. However, most of these studies focus on flat leaf surfaces and overlook the more essential geometric features of real plant leaves. On the one hand, the curvature distribution from the leaf base to the leaf tip exhibits pronounced spatial variation [11]. On the other hand, under external disturbances, the bending deformation of leaves leads to additional changes in curvature, resulting in dynamic geometric evolution [12]. The influence of these geometric characteristics on droplet impact behavior has not yet received systematic attention.

A limited number of studies have indicated that curvature can break the axisymmetric spreading of droplets and induce asymmetric spreading, fragmentation, or rebound [13]– [14]. Similar tendencies have been verified using artificial cylindrical and spherical models [15]– [16]. More recently, research has expanded toward dynamic surface characteristics, highlighting that vibration introduces additional inertial effects to impacting droplets and consequently alters their spreading dynamics and splashing thresholds [17]– [18]. Nevertheless, such studies often employ idealized or artificially imposed vibration parameters that differ substantially from the natural vibration behavior of real crop leaves subjected to airflow and other environmental forces. Recent work by Cao et al. [19]– [20], combining numerical simulations and experiments, has further demonstrated that leaf vibration induced by mechanical excitation can enhance droplet spreading and deposition.

In summary, most existing studies have focused either on the intrinsic properties of droplets or on individual leaf-related factors such as curvature or vibration. However, a systematic investigation of droplet impact behavior under the coupled effects of realistic leaf curvature and vibration is still lacking. To address this gap, the present study uses capsicum leaves, a representative crop in field conditions, and incorporates their actual dynamic characteristics obtained from wind tunnel experiments, including curvature distribution and frequency response. We systematically elucidate the coupled mechanisms among Weber number (*We*), leaf curvature (C^*^), and vibration frequency (*f*), and further develop a predictive model for the maximum spreading diameter as well as a quantitative method for evaluating droplet detachment risk. The findings of this study enhance the understanding of droplet-leaf interaction mechanisms and provide valuable guidance for optimizing spraying parameters and improving the efficiency of pesticide application.

## Materials and methods

### Acquisition of leaf physical parameters

The single-hanging-track air-assisted sprayer [21] has been widely adopted in sunlight greenhouses in Shouguang City, China, due to their high efficiency and minimal floor space requirements. However, operating with a uniform wind speed often causes uneven droplet deposition on crop leaves, leading to double spraying, missed spraying, and insufficient pesticide coverage. Therefore, improving airflow control is essential for precise application.

**Fig. 1:**
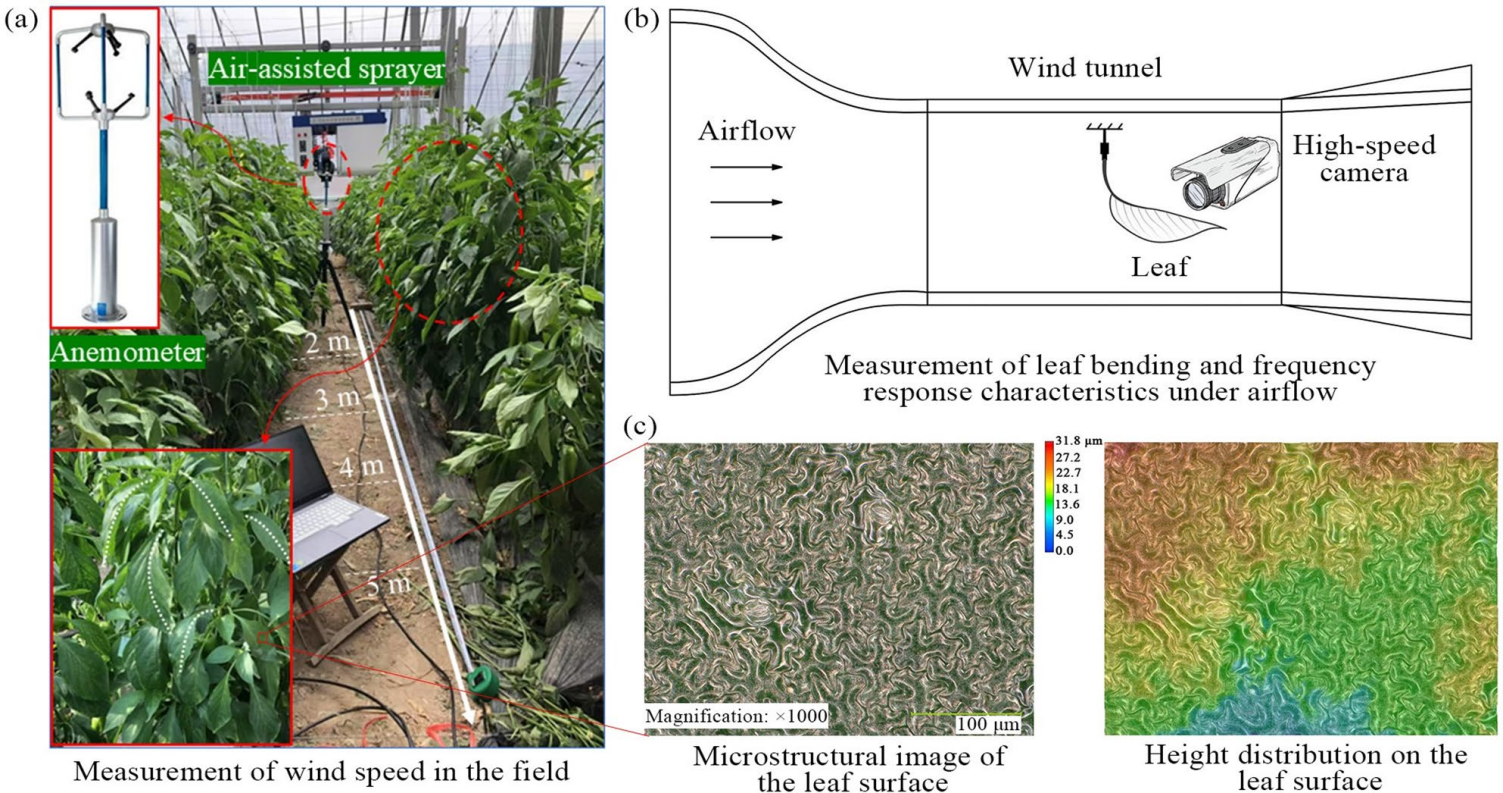
Acquisition of leaf physical parameters: (**a**) field wind speed measurement; (**b**) acquisition of leaf aerodynamic response; (**c**) surface microstructure of capsicum leaves

Field measurements with a three-dimensional ultrasonic anemometer (Wind Master Pro, Gill Instruments Ltd., UK) showed wind speeds within 1–5 m of crop rows ranging from 2.4 to 11.3 m/s (Fig. 1a). During actual spraying, the leaves undergo further deformation on top of their natural curvature, accompanied by oscillation. To clarify these dynamics, leaf aerodynamic responses at different wind speeds were studied under controlled conditions using a wind tunnel and high-speed imaging system (Fig. 1b), following methods described in previous studies [12, 22]. Results showed that below 9 m/s, the leaves respond to aerodynamic forces based on a geometrically curved configuration, with a curvature coefficient less than 0.101 mm^–1^ and oscillation frequencies < 30 Hz. Above 9 m/s, this curved morphology disappeared in video imaging as leaves mainly underwent nonlinear deformations such as flipping.

To investigate the impact dynamics of droplets on leaf surfaces, intact and healthy capsicum leaves were selected as experimental specimens. Fresh capsicum plants (variety: Tianshuai 101) with a growth period of approximately 5 months were collected in November 2024 from the Sunjia Village Family Farm, located in Danyang City, Jiangsu Province, China. Three-dimensional reconstruction images of the leaf surface microstructure were acquired using a high-depth-of-field three-dimensional microscope (VHX-900 F, Keyence Corporation, Japan), and surface height distribution maps were generated (Fig. 1c). Based on these height data, the arithmetic mean roughness (*Ra*) was calculated. The static contact angle of the leaf surface was measured using a contact angle meter (OCA 25, Dataphysics, Germany). Given that the edge contour of capsicum leaves is approximately elliptical, leaf length (*L*) and width (*W*) were used to characterize their geometric dimensions. The physical parameters of the leaves are summarized in Table 1.

**Table 1:** Physical parameters of the leaf

Parameter	Unit	Value
Leaf length (*L*)	mm	61.1 ± 4.6
Leaf width (*W*)	mm	28.4 ± 2.1
Leaf curvature (*κ*)	mm^–1^	≤ 0.1
Leaf vibration frequency (*f*)	Hz	< 30
Static contact angle (CA)	°	79.3 ± 6.4
Surface roughness (*Ra*)	µm	6.5 ± 2.2

Before the experiment, leaf segments measuring 14 mm × 14 mm were excised from the leaf surface using scissors. The substrate curvature was determined from measured leaf curvatures under wind loading. Previous research [12] showed that overall leaf curvature remains below 0.101 mm^–1^, with deformation dominated by the leaf base at low wind speeds (< 6 m/s; curvature < 0.031 mm^–1^) and by the leaf tip at higher speeds (> 6 m/s), where curvature increases markedly. To capture representative bending states while avoiding extreme tip effects, a curvature range of 0–0.055 mm^–1^ was selected for substrate fabrication. Substrates were made of nylon fiber to ensure controllable, stable curvature. Leaf sections were attached smoothly without wrinkles, and the process was completed within one minute to minimize moisture loss. Fresh leaf samples were used for each trial to ensure accuracy and reproducibility.

### Overall experiment setup

**Fig. 2:**
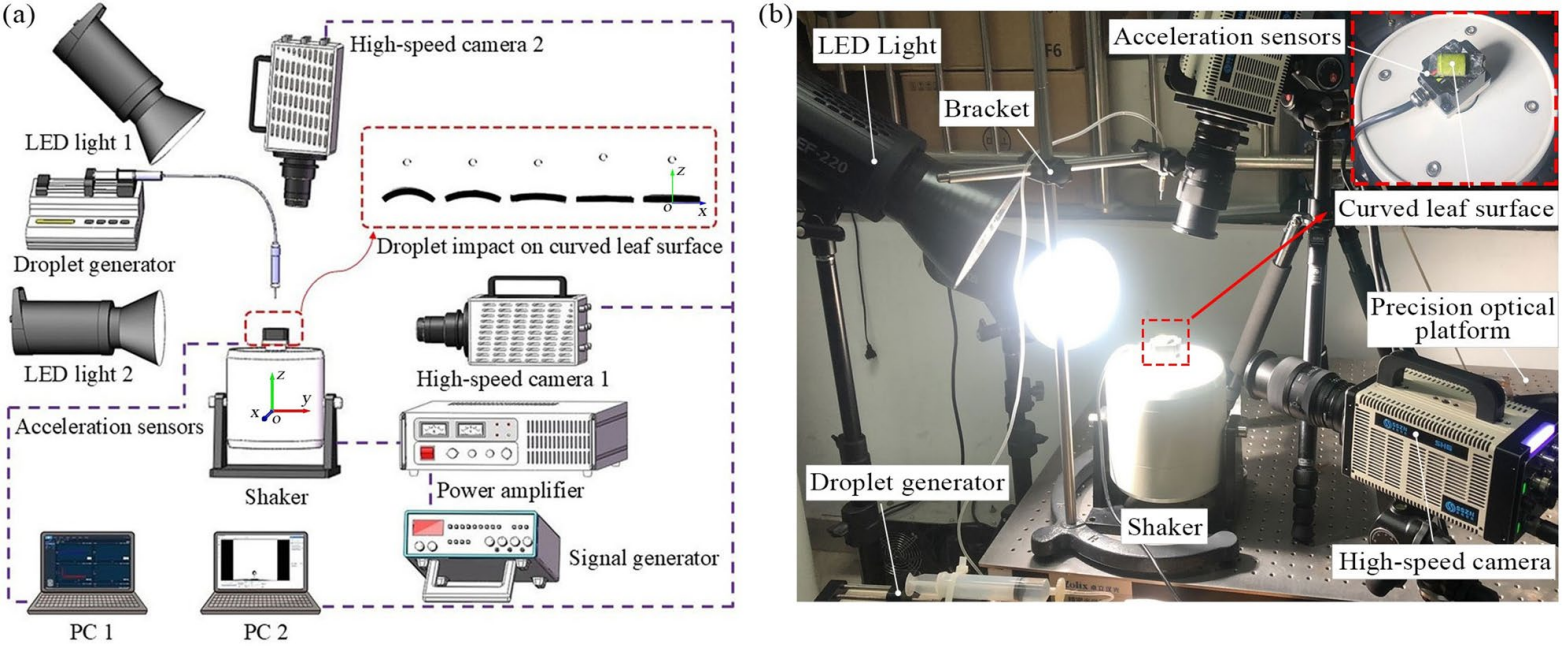
Overview of the experimental setup: (**a**) schematic diagram; (**b**) photograph of the setup

The experimental setup consisted of three subsystems: a droplet generation system, a leaf vibration system, and an image acquisition system (Fig. 2). The droplet generation system comprised a micro syringe pump (MDG-100, TSI, USA), a hose, a syringe, and a needle. The leaf vibration system included an exciter (JZK-20, SINOCERA, China), a signal generator (4011 A, BK PRECISION, USA), a power amplifier (YE5873A, SINOCERA, China), a three-dimensional acceleration sensor (WT-VB02-485, Shenzhen Witte Intelligent Technology Co., Ltd., China), a precision optical table (ZOLIX, Beijing, China), and a curved substrate.

To clearly describe spatial distribution, an *o*-*xyz* coordinate system was established with the origin at the exciter center: the *y*-axis was aligned with the lateral direction of the exciter’s output shaft, the *x*-axis followed the right-hand rule, and the *z*-axis was perpendicular to the horizontal plane and coincident with the shaft axis. The image acquisition system consisted of two high-speed cameras (SH-6-109, Shenzhen SinceVision Technology Co., Ltd., China), two LED light sources, and two laptops for synchronized imaging and data storage. The cameras were arranged along the *y*- and *z*-axes, respectively. Due to obstruction by the droplet generation system, the *z*-axis camera was mounted at a slight angle, while the *y*-axis camera was positioned opposite an LED light source for backlit imaging.

### Experimental methods for leaf surface droplet dynamics

#### Droplet parameter dimensionless

In field pesticide spraying, droplets often have high Weber numbers, making them prone to severe deformation or breakup and increasing uncertainty in observations. Moreover, limits in high-speed camera resolution and needle precision hinder direct generation of micron-scale droplets identical to field conditions. To address this, we applied hydrodynamic similarity theory, using dimensionless numbers to ensure comparable droplet dynamics between lab and field. Specifically, the Weber (*We*), Reynolds (*Re*), and Ohnesorge (*Oh*) numbers describe the relative effects of inertia, surface tension, and viscosity, and are the key governing parameters for droplet impact on leaves [23]. In addition, a dimensionless leaf curvature (C^*^) is defined to represent leaf bending, while the Bond number (*Bo*) evaluates the effects of droplet size and inertial forces from leaf vibration.

1 $$\begin{aligned} We=&\frac{{\rho {v^2}{D_0}}}{\sigma },\operatorname{Re} =\frac{{\rho v{D_0}}}{\mu },\\&Oh=\frac{\mu }{{\sqrt {\rho \sigma {D_0}} }}=\frac{{W{e^{\frac{1}{2}}}}}{{\operatorname{Re} }},\\&{C^*}=\frac{{{D_0}}}{R}={D_0} \cdot \kappa \\ \\&B{o_g}=\frac{{\rho {D_0}^{2}g}}{\sigma },\\&B{o_i}=\frac{{\rho {D_0}^{2}L{\omega ^2}}}{{2\sigma }} \end{aligned}$$

Where *We* is the Weber number; *ρ* is the liquid density, kg/m^3^; *v* is the droplet impact velocity, m/s; *D*_0_ is the droplet diameter, m; *σ* is the surface tension, N/m; *Re* is the Reynolds number; *µ* is the dynamic viscosity of the droplet, Pa·s; *Oh* is the Ohnesorge number; C^*^ is the dimensionless leaf curvature; *R* is the radius of leaf curvature, m; *κ* is the leaf curvature, m^–1^; *Bo*_*g*_ is the Bond number considering the effect of gravity; *g* is gravitational acceleration, m/s^2^; *Bo*_*i*_ is the Bond number considering the effect of leaf inertial force; *L* is the length of the leaf, m; *ω* is the oscillation angular frequency of the leaf, rad/s, *ω* = 2*πf*; *f* is the oscillation frequency of the leaf, Hz.

In field pesticide spraying, surfactants are often added to reduce surface tension and enhance droplet spreading and adhesion on leaves [24]. Under such conditions, spray droplets typically have surface tensions of 0.0194–0.064 N/m, viscosities of 0.001–0.1 Pa·s, diameters of 147–1138 μm, and impact velocities below 22.27 m/s [25]. In this study, droplets with an initial diameter of *D*_0_ = 2.003 × 10⁻^3^ m were used. Based on Eq. (1), the corresponding field-relevant dimensionless ranges are *We* = 2.3–29,500, *Re* = 1.47–25,300, and *Oh* = 0.0037–1.873. Although exact similarity across all three parameters is challenging, the experiments realistically simulate low-to-medium velocity and low-viscosity spray conditions. When *We* ≤ 250, surface tension effectively suppresses excessive deformation or breakup [23], ensuring stable observations. To achieve this, a 0.1% Tween 80 aqueous solution was prepared (1 g Tween 80 dissolved in 999 g deionized water, stirred for 30 min). The solution had a surface tension of 0.04 N/m (pendant drop method, error < 2%), with density (1000 kg/m^3^) and viscosity (0.001 Pa·s) close to water, thereby covering the Reynolds number range of field droplets while approximating their Ohnesorge numbers.

In summary, substituting parameters such as *We*, *ρ*, and *D*_0_ into Eq. (1) and constraining the droplet impact velocity to *v* ≤ 2 m/s yields *Re* < 4006, *Oh* ≈ 0.0112, and a maximum *Bo*_*g*_ value of 0.98 (< 1). This indicates that droplet dynamics are dominated by surface tension, with gravitational effects being negligible. In contrast, when accounting for inertial forces generated by leaf oscillation, Table 1 shows that for leaves with length *L* < 0.07 m and oscillation frequency *f* < 30 Hz under low wind speeds (≤ 9 m/s), the maximum *Bo*_*i*_ value reaches 124.6 (> 1). This demonstrates that droplet behavior is strongly influenced by inertial forces arising from leaf oscillation. Therefore, this study emphasizes the role of leaf oscillation in shaping droplet impact behavior, aiming to provide deeper insights into droplet–leaf interactions during pesticide spraying.

#### Droplet impact experiment on static leaf surfaces

To investigate droplet impact behavior on a static leaf surface, a curved substrate was mounted at the center of the exciter’s output shaft, with the leaf axis aligned along the *y*-axis and its surface normal oriented as parallel as possible to the *z*-axis (Fig. 2). In this configuration, the exciter was kept de-energized to ensure that the leaf surface remained stationary. In the droplet generation system, the syringe was connected to the needle through a flexible hose, allowing the needle to be positioned precisely above any target location on the leaf surface. The droplet impact velocity was controlled by adjusting the vertical distance between the needle tip and the leaf surface. A micro-syringe pump delivered liquid at a flow rate of 50 mL/h, producing stable, individual droplets that detached under gravity and impacted the surface vertically.

**Fig. 3:**
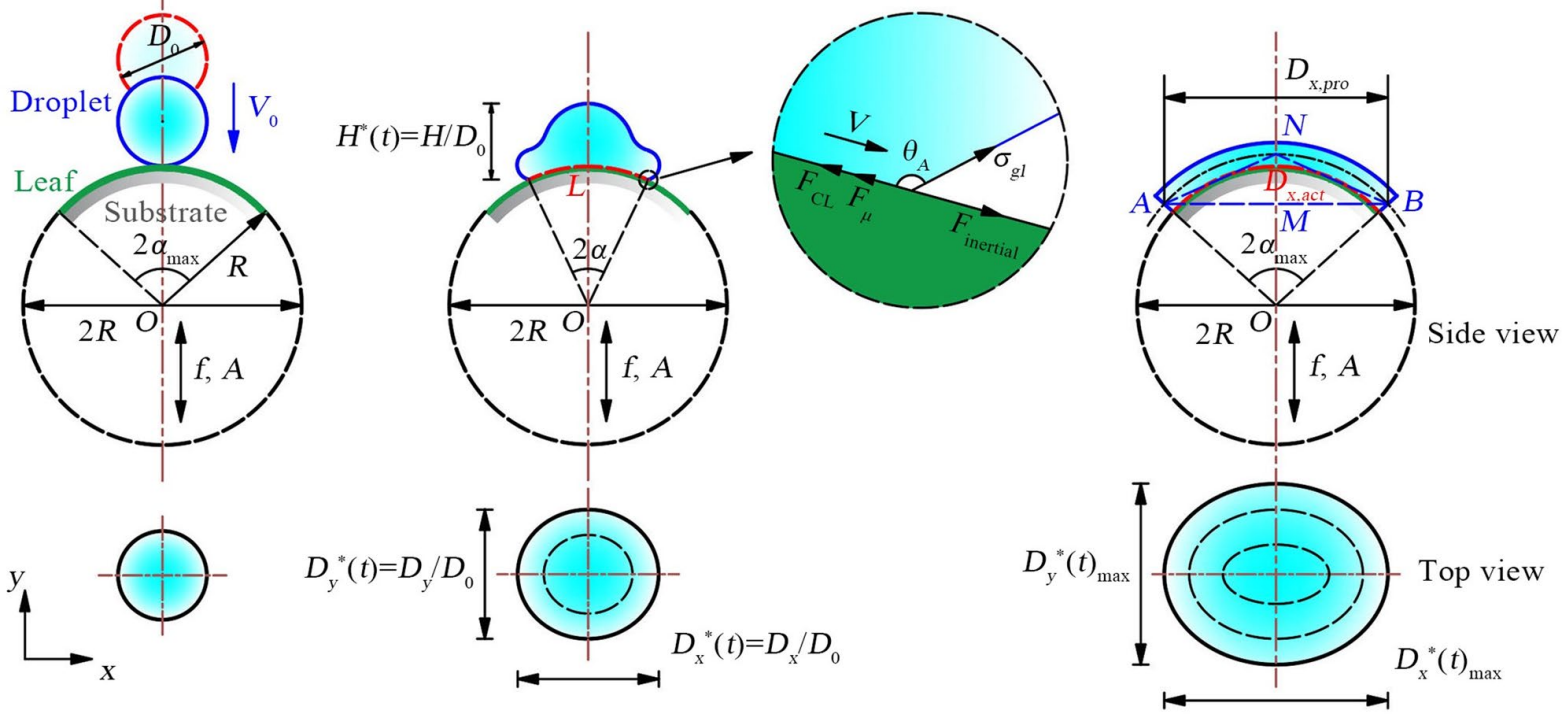
Schematic diagram of the droplet impact process

Figure 3 shows the droplet impact process on the curved leaf surface, consisting of initial contact, spreading, and maximum spreading. Two high-speed cameras recorded the impact synchronously from orthogonal directions, enabling simultaneous acquisition of side- and top-view images for reconstructing the droplet’s three-dimensional dynamics. Before testing, a calibration plate was placed on the leaf surface for pixel-length conversion, and lens distortion was corrected. The imaging settings were 5000 fps and 512 × 288 pixels. The videos were processed using ProAnalyst software (Xcitex, Inc., Woburn, MA, USA) to extract key parameters, including the spreading diameters *D*_x_^*^ and *D*_y_^*^, the maximum spreading diameters *D*_x_^*^_max_ and *D*_y_^*^_max_, the droplet height H^*^, and the spreading and retraction times *t*_1_ and *t*_2_.

To ensure that these parameters reliably represent droplet dynamics, the projection errors introduced by leaf curvature must be considered. The droplet footprint on the curved surface is a chord, denoted as *AB*, corresponding to the two-dimensional projected spreading diameter *D*_*x*,pro_ captured by the camera. In contrast, the actual spreading path on the leaf surface is an arc, *D*_*x*,act_. According to the geometric relationships shown in Fig. 3:

2 $$\left\{ \begin{gathered} \sin {\alpha _{\hbox{max} }}=\frac{{AM}}{{OA}}=\frac{{{D_{x,pro}}}}{{2R}} \hfill \\ \cos {\alpha _{\hbox{max} }}=\frac{{AM}}{{AN}}=\frac{{{D_{x,pro}}}}{{{D_{x,act}}}} \hfill \\ \end{gathered} \right.$$

Where *α* is the central angle of the semicircle corresponding to droplet spreading, °; *D*_*x*,pro_ is the two-dimensional projected value of droplet spreading, m; *D*_*x*,act_ is the actual value of droplet spreading, m.

Using the most extreme condition in this study (C^*^ = 0.055, *We* = 168) as an example, based on Eq. (2), the difference between *D*_*x*,act_ and *D*_*x*,pro_ was only 0.13%, indicating minimal projection distortion. Since *D*_y_^*^ and H^*^ correspond to spreading along the leaf’s axial direction (*y*-axis) and deformation along the surface normal (*z*-axis), no two-dimensional projection error occurs. Thus, projection effects on these parameters are negligible, confirming that they reliably characterize droplet spreading, retraction, and stabilization on curved leaf surfaces.

In this study, droplets are sufficiently small (*Bo*_*g*_ < 1), so gravity can be neglected. Consequently, the droplet’s maximum spreading after impact is governed primarily by inertial forces, contact-line forces, and viscous resistance. During outward spreading the droplet decelerates from its initial velocity to an instantaneous stop at the contact line. Considering a small fluid segment near the contact line as the control volume (Fig. 3), its motion obeys Newton’s second law. The local force balance along the spreading direction is:

3 $${F_{CL}}+{F_\mu }={F_{inertia}}=m\dot {V}$$

Where *m* is the effective mass of the liquid involved in spreading, Kg; *V* is the droplet spreading velocity, m/s; *F*_*CL*_ is the contact line force, N; *F*_*µ*_ is the viscous resistance at the solid-liquid interface, N; *F*_*inertia*_ is the inertial force associated with droplet spreading, N.

As a droplet spreads on a curved leaf, the contact line is deflected by the local geometry, which reduces the effective surface-tension projection along the spreading direction. This curvature therefore suppresses the contact-line driving force. To quantify the reduction, a curvature-dependent correction function Г(C^*^) is introduced. It satisfies Г(0) = 1 for a flat surface and decreases monotonically with increasing curvature. The contact-line force is then written as:

4 $${F_{CL}}={\sigma _{gl}}L\cos {\theta _A}\Gamma \left( {{C^*}} \right)$$

Where *σ*_*gl*_ is the surface tension between liquid and gas, N/m; *L* is the contact line length, m; *θ*_*A*_ is the dynamic contact angle, °.

At the instant when the droplet reaches its maximum spreading, its velocity decreases to zero, but the acceleration is not zero. By approximating the acceleration as $\dot {V}\sim {{ - V_{0}^{2}} \mathord{\left/ {\vphantom {{ - V_{0}^{2}} {{D_{\hbox{max} }}}}} \right. \kern-0pt} {{D_{\hbox{max} }}}}$, and substituting it into Eq. (3), the relationship for the maximum spreading diameter can be obtained:

5 $${D_{\hbox{max} }}\sim \frac{{mV_{0}^{2}}}{{{\sigma _{gl}}L\cos {\theta _A}\Gamma \left( {{C^*}} \right)+{F_\mu }}}$$

Where *D*_max_ is the maximum spreading diameter of the droplet, m; *V*_0_ is the initial impact velocity of the droplet, m/s.

By combining Eq. (1), which defines the Weber number, and considering that the droplet parameters in this study are fixed, Eq. (5) can be simplified as follows:

6 $${D_{\hbox{max} }}\sim \frac{{We}}{{L\cos {\theta _A}\Gamma \left( {{C^*}} \right)+\left( {{{{F_\mu }} \mathord{\left/ {\vphantom {{{F_\mu }} {{\sigma _{gl}}}}} \right. \kern-0pt} {{\sigma _{gl}}}}} \right)}}$$

When extended to the case of leaf vibration, where the leaf undergoes harmonic motion with frequency *f* and amplitude *A*, the maximum droplet spreading diameter can be further adjusted as follows:

7 $$\left\{ \begin{gathered} {D_{\hbox{max} }}\sim \frac{{W{e_{vib}}}}{{L\cos {\theta _A}\Gamma \left( {{C^*}} \right)+\left( {{{{F_\mu }} \mathord{\left/ {\vphantom {{{F_\mu }} {{\sigma _{gl}}}}} \right. \kern-0pt} {{\sigma _{gl}}}}} \right)}} \\ \begin{array}{*{20}{c}} {W{e_{vib}}=\frac{{\rho {D_0}V_{{eff}}^{2}}}{{{\sigma _{gl}}}},}&{{V_{eff}}={V_0}+\left( {2\pi f} \right)A\cos {\theta _p}} \end{array} \\ \end{gathered} \right.$$

Where *We*_*vib*_ is the vibration Weber number; *V*_*eff*_ is the effective impact velocity of the droplet, m/s; *θ*_*p*_ is the phase difference between the droplet and the leaf.

Due to the geometric effects of the curved leaf surface, droplet spreading becomes asymmetric. An asymmetry factor, *λ*, is introduced to quantify the difference in spreading along the *x* and *y* directions. This leads to the final relationship for the maximum spreading diameter of the droplet in different directions:

8 $$\left\{ \begin{gathered} \begin{array}{*{20}{c}} {{D_{x,\hbox{max} }} \approx \frac{{W{e_{vib}} \cdot \lambda }}{{L\cos {\theta _A}\Gamma \left( {{C^*}} \right)+\left( {{{{F_\mu }} \mathord{\left/ {\vphantom {{{F_\mu }} {{\sigma _{gl}}}}} \right. \kern-0pt} {{\sigma _{gl}}}}} \right)}},}&{{D_{y,\hbox{max} }} \approx \frac{{{D_{x,\hbox{max} }}}}{\lambda }} \end{array} \\ \begin{array}{*{20}{c}} {\lambda =\frac{{{D_{x,\hbox{max} }}}}{{{D_{y,\hbox{max} }}}},}&{\lambda \geqslant 1} \end{array} \\ \end{gathered} \right.$$

Based on Eq. (8), the effects of *We* and C^*^ on the maximum spreading diameter can be systematically evaluated. As *We* (or *We*_*vib*_) increases, inertial forces become increasingly dominant over surface tension and viscous effects, leading to larger *D*_*x*,max_ and *D*_*y*,max_. Leaf curvature C^*^ influences spreading through the curvature-correction function Г(C^*^) and the asymmetry factor *λ*. Increasing C^*^ reduces Г(C^*^) and elevates *λ*, causing *D*_*x*,max_ to grow more rapidly than *D*_*y*,max_ and thereby amplifying spreading asymmetry.

To isolate these effects, controlled experiments will be performed by varying the impact velocity to adjust *We* and selecting substrates of different curvature to tune C^*^. These tests will provide empirical validation of the predicted trends and offer guidance for regulating droplet impact behavior.

#### Experiments on droplet dynamics on vibrating surfaces

To investigate the influence of leaf vibration on droplet dynamics, experiments were conducted on droplets deposited on vibrating leaf surfaces. Before the tests, the shaker was firmly mounted on a precision optical platform to ensure system stability. A three-dimensional accelerometer was attached to the shaker’s output shaft to monitor real-time vibration frequency and amplitude. The leaf was then fixed at the accelerometer center using double-sided tape, ensuring that its vibration remained stable and synchronized with the shaker.

**Fig. 4:**
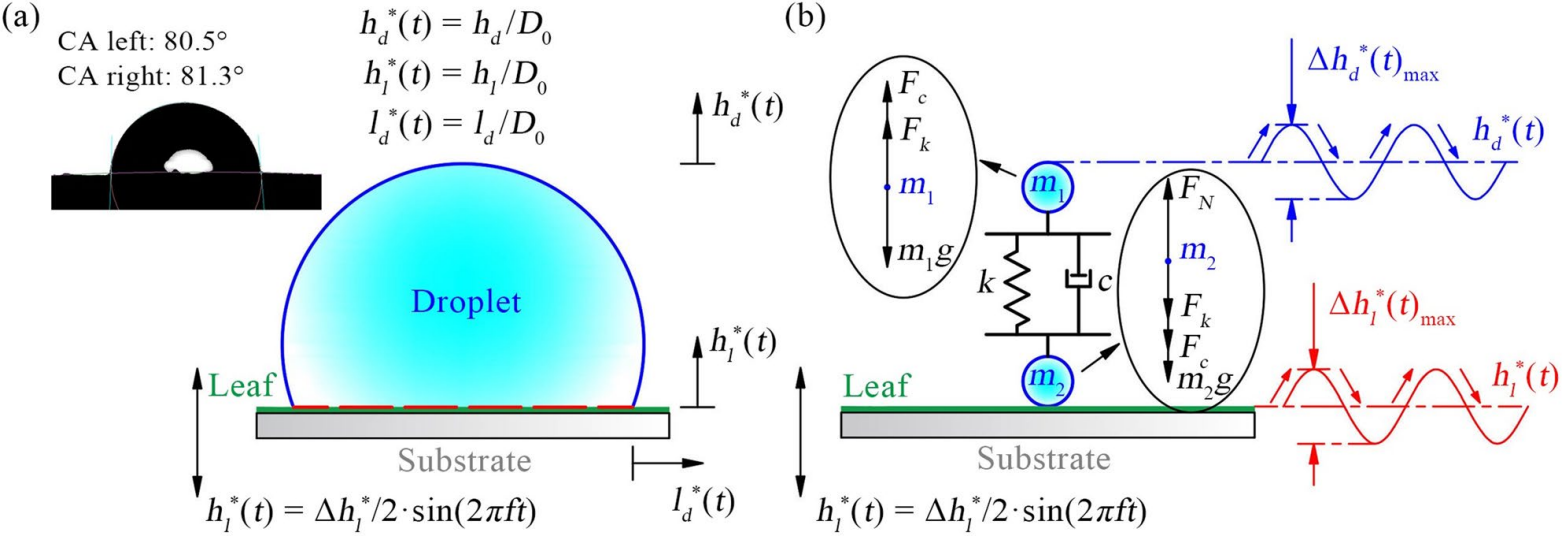
Schematic diagram of droplet parameters: (**a**) droplet displacement; (**b**) forced vibration model

During the experiments, sinusoidal signals in the 10–80 Hz frequency range were generated by a signal generator, amplified through a power amplifier, and subsequently delivered to the shaker, inducing vertical oscillations of the leaf at the prescribed frequency. Droplets were gently deposited from a needle onto the leaf surface prior to vibration onset, thereby avoiding impact effects. The subsequent droplet dynamics were simultaneously recorded by two high-speed cameras from orthogonal viewing angles, enabling comprehensive observation of oscillation, spreading, and contraction processes. To quantitatively characterize droplet motion, three dimensionless displacement parameters were defined: the vertical displacement of the droplet apex *h*_d_^*^(*t*), the lateral displacement at the droplet contact point *l*_d_^*^(*t*), and the vertical displacement of the leaf surface *h*_l_^*^(*t*), as shown in Fig. 4a.

To further investigate the dynamics of droplets on vibrating leaves, the droplet was modeled as a two-mass spring-damper system [26], consisting of a top mass *m*_1_ and a bottom mass *m*_2_, as illustrated in Fig. 4b. In this model, the droplet surface tension is represented by a spring with stiffness *k*, and the droplet viscosity is represented by a damper with damping coefficient *c*. For the top mass *m*_1_, the force balance equation in the vertical direction is given by:

9 $$\left\{ \begin{gathered} {m_1}{{\ddot {h}}_d}\left( t \right)+{F_c}+{F_k} - {m_1}g=0 \hfill \\ {F_c}=c\left( {{{\dot {h}}_d}\left( t \right) - {{\dot {h}}_l}\left( t \right)} \right) \hfill \\ {F_k}=k\left( {{h_d}\left( t \right) - {h_l}\left( t \right)+\varepsilon } \right) \hfill \\ \end{gathered} \right.$$

Where *F*_*c*_ is the damping force, N; *F*_*k*_ is the equivalent elastic force, N; *ε* is the pre-compression amount at the initial equilibrium of the droplet, m.

For the bottom mass *m*_2_, which remains adhered to the leaf and moves synchronously with it, the force balance equation is given by:

10 $${m_2}{\ddot {h}_l}\left( t \right) - {F_c} - {F_k} - {m_2}g+{F_N}=0$$

Before the leaf begins to vibrate, the droplet is at rest, and the top mass *m*_1_ is in equilibrium under the balance of gravity and elastic forces, which can be expressed as:

11 $$k\varepsilon - {m_1}g=0$$

Since the bottom mass *m*_2_ moves synchronously with the leaf, its displacement is directly equivalent to the leaf displacement, expressed as:

12 $${h_l}\left( t \right)=A\sin \left( {\omega t} \right)=A\sin \left( {2\pi ft} \right)$$

By solving Eqs. (9), (11), and (12) simultaneously, the governing equation for the top mass *m*_1_ can be obtained as:

13 $$\begin{aligned}{m_1}{\ddot {h}_d}\left( t \right)&+c{\dot {h}_d}\left( t \right)+k{h_d}\left( t \right)\\&=kA\sin \left( {\omega t} \right)+c\omega A\cos \left( {\omega t} \right)\end{aligned}$$

Based on Eq. (13), the droplet amplitude amplification factor can be further derived as:

14 $$\varphi =\frac{{\sqrt {1+4{\xi ^2}{\lambda ^2}} }}{{\sqrt {{{\left( {1 - {\lambda ^2}} \right)}^2}+{{\left( {2\xi \lambda } \right)}^2}} }}$$

Where *λ* is the frequency ratio, $\lambda =\frac{\omega }{{{\omega _n}}}$; *ωₙ* is the natural frequency of the system; *ξ* is the damping ratio, $\xi =\frac{c}{{2\sqrt {k{m_1}} }}=\frac{c}{{2{m_1}{\omega _n}}}$.

The phase difference between the droplet and the leaf motion, *θₚ*, is given by:

15 $$\tan {\theta _p}=\frac{{2\xi {\lambda ^3}}}{{1 - {\lambda ^2}+4{\xi ^2}{\lambda ^2}}}$$

According to Eq. (14), when *λ* ≈ 1, *φ* increases significantly, and the droplet top’s amplitude greatly exceeds that of the leaf, indicating that the system enters a resonance state. Equation (15) further shows that for *λ* < 1, *θₚ* is small, suggesting that the droplet and leaf move approximately in sync; for *λ* ≈ 1, *θₚ* jumps toward *π*/2; and for *λ* > 1, *θₚ* approaches *π*, indicating that the droplet and leaf move in opposite directions.

To validate these predictions and clarify the effect of leaf vibration on droplet dynamics, experiments will systematically measure droplet amplitude and phase shift across different frequencies. Comparing these results with theory allows identification of the resonance region and quantification of vibration effects on spreading and detachment, thereby testing the model and refining the droplet-vibrating leaf interaction mechanism.

#### Multi-factor orthogonal experiment

To systematically elucidate the coupled effects of multiple factors on droplet impact dynamics, a multifactor orthogonal experimental design was developed based on single-factor tests conducted on both static and vibrating leaf surfaces. The study focused on three primary parameters: the droplet impact parameter (Weber number, *We*), the leaf geometric parameter (dimensionless curvature, C^*^), and the leaf vibration parameter (vibration frequency, *f*). A three-factor, three-level Box-Behnken central composite design was adopted, consisting of 17 experimental runs. The factor levels were determined from the results of preliminary single-factor experiments, as summarized in Table 2.

**Table 2:** Experimental factors and levels

Factor	Levels		
	−1	0	1
*We*	128	148	168
*C* ^*^	0.013	0.027	0.041
*f*	40	45	50

To minimize random errors, each experimental run was independently repeated ten times, resulting in a total of 170 tests. The sequence of the 17 basic runs was randomized using a random number table. For each test, a fresh capsicum leaf segment was used. Data points where droplets failed to strike the curved surface center or impacted the leaf vein were excluded (accounting for less than 5% of the total), and the average value of valid repetitions was taken as the final response under each condition.

The dimensionless maximum spreading diameter (defined as the ratio of the droplet’s maximum spreading diameter to its initial diameter, denoted D^*^_max_) was selected as the response variable. Quadratic regression and response surface analyses were performed using Design Expert 8.0.6 software to establish a second-order polynomial model of D^*^_max_ as a function of *We*, C^*^, and *f*. Model reliability was evaluated through analysis of variance (ANOVA) to assess its significance and goodness of fit. Finally, response surface plots were generated to visually illustrate the individual and interactive effects of the key factors on droplet spreading behavior.

## Results and discussion

### Impact results of the droplet on static leaf surfaces

**Fig. 5:**
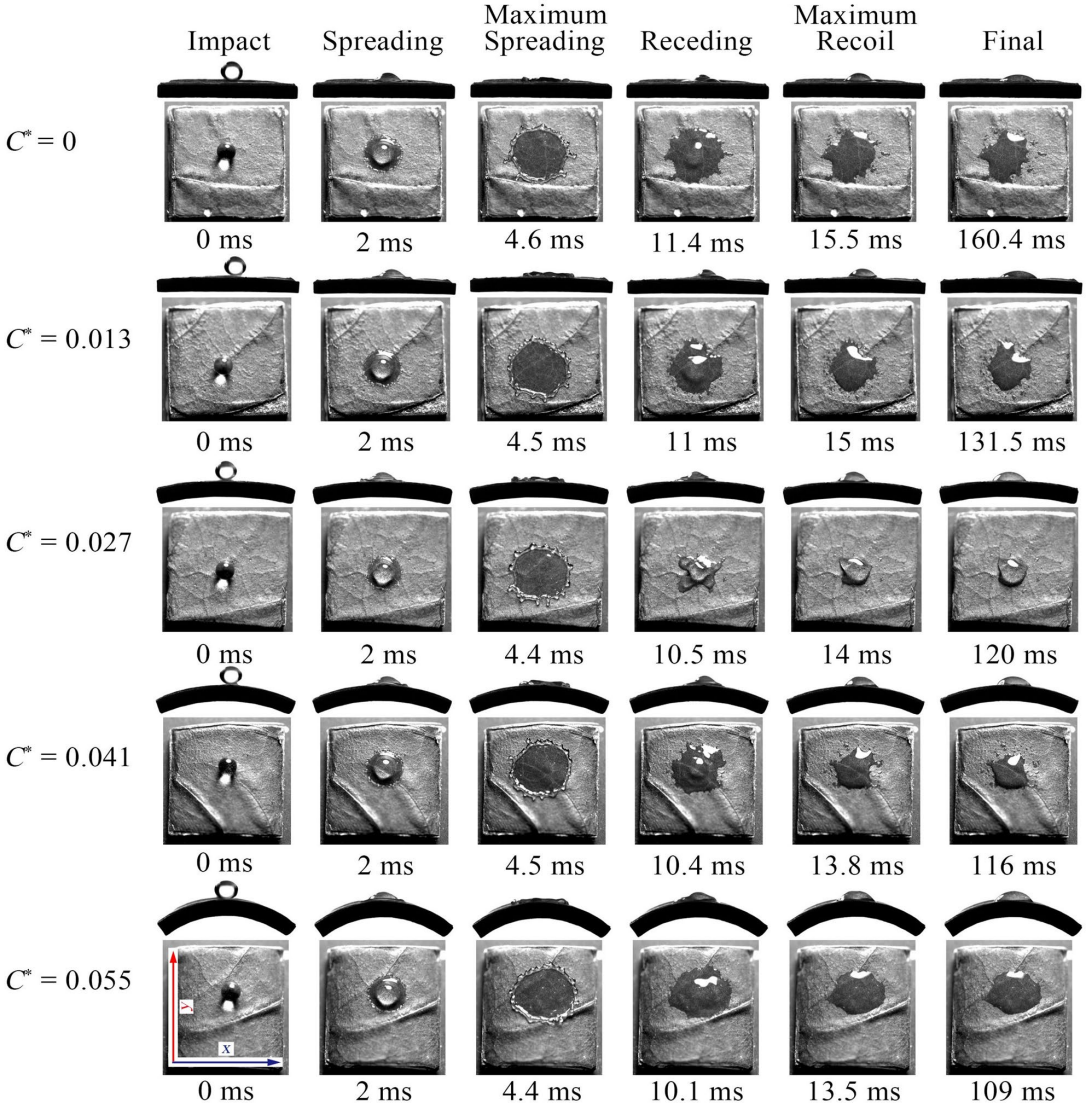
Droplet impact behavior corresponding to different C^*^ at *We* = 128

Figure 5 presents the droplet impact behavior at different C^*^ values under *We* = 128, illustrating the sequential stages of initial contact, spreading, maximum spreading, retraction, and final stabilization. The results indicate that the maximum spreading morphology transitions from circular to elliptical as C^*^ increases. This shift arises from geometric constraints during impact on curved surfaces, which induce anisotropic kinetic energy distribution [13]. Specifically, the curvature generates tangential momentum that promotes preferential spreading along the *x*-axis while enhancing energy dissipation along the *y*-axis. Consequently, an elliptical spreading pattern emerges, with *D*_x_^*^_max_ notably exceeding *D*_y_^*^_max_.

During retraction, the elliptical residual effect at high C^*^ delays the restoring action of surface tension, yielding a slightly elliptical stable state. In contrast, droplets at low C^*^ rapidly retract into near-circular shapes. Furthermore, local variations in leaf surface microstructure or wettability can destabilize the liquid film, producing satellite droplets that lead to irregular final morphologies. Such instabilities are evident on both flat and low C^*^ surfaces.

#### Asymmetric droplet spreading induced by leaf surface curvature

**Fig. 6:**
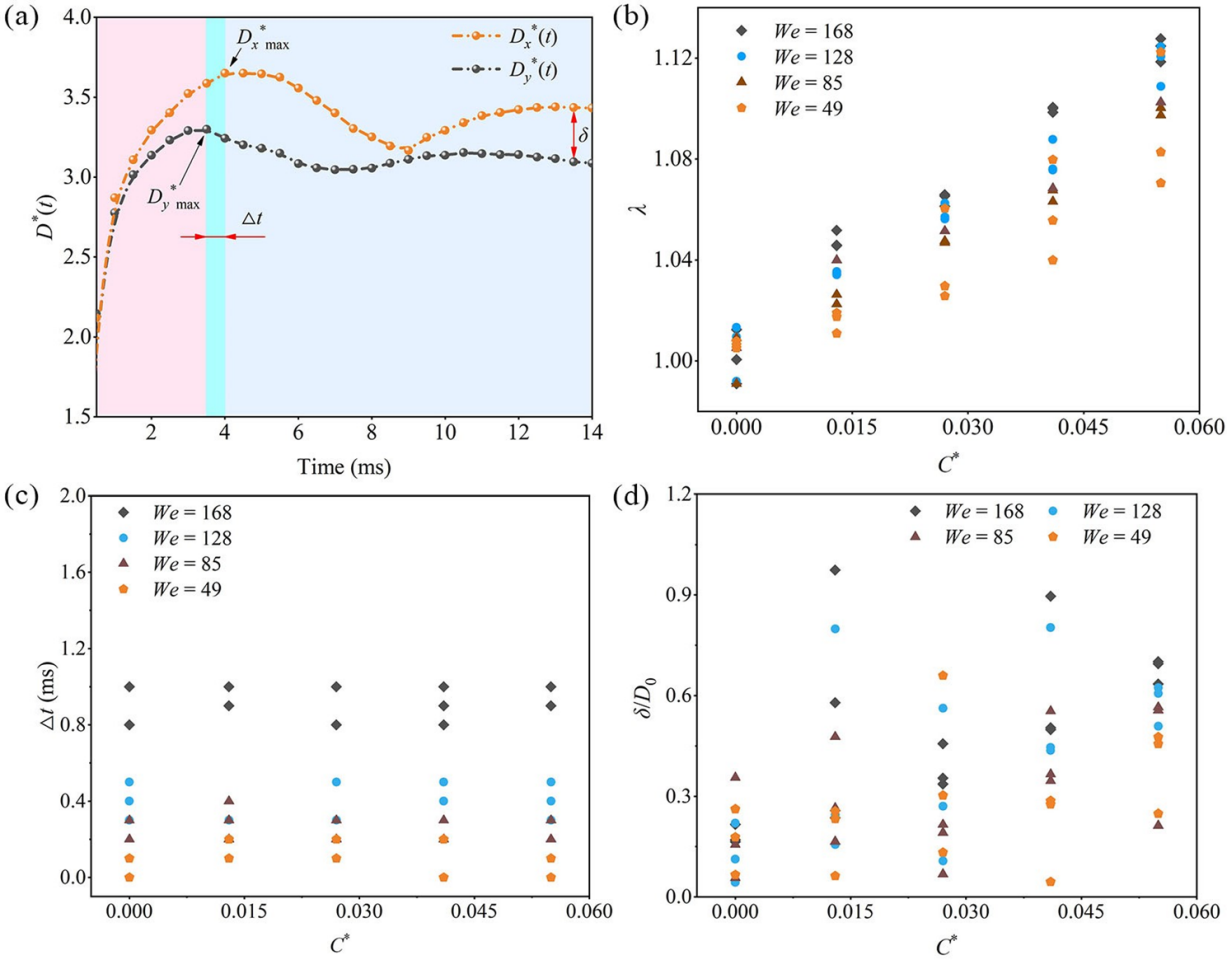
Influence of *We* and C^*^on droplet asymmetric spreading: (**a**) D^*^(*t*); (**b**) *λ*; (**c**) Δ*t*; (**d**) *δ*

To further validate and quantify the asymmetric spreading behavior of droplets on leaf surfaces with different C^*^ values, impact experiments were performed under varying *We* and C^*^ conditions. Figure 6a shows the temporal evolution of spreading diameters *D*_x_^*^ and *D*_y_^*^ for droplets at *We* = 128 and C^*^ = 0.055. The results indicate that *D*_y_^*^ reaches its maximum at *t* = 3.5 ms, whereas *D*_x_^*^ continues to increase and peaks at *t* = 4 ms. This highlights a clear discrepancy between *D*_x_^*^ and *D*_y_^*^ during the spreading process.

To quantify this asymmetry, three parameters were defined based on Fig. 6a: (i) the asymmetry factor *λ*, expressed as the ratio of *D*_x_^*^_max_ to *D*_y_^*^_max_; (ii) the asymmetry time difference Δ*t*, defined as the time lag between *D*_x_^*^_max_ and *D*_y_^*^_max_; and (iii) the residual asymmetry *δ*, representing the difference between *D*_x_^*^ and *D*_y_^*^ at the final steady state. The corresponding results are presented in Fig. 6b-d.

Figure 6b shows that when C^*^ = 0 (flat surface), *λ* ≈ 1, indicating nearly symmetric droplet spreading. As C^*^ increases, *λ* rises significantly; for *We* = 49 and 168, C^*^ = 0.055 increases *λ* by 8.49% and 12.21%, respectively, compared with a flat surface. This is attributed to curvature-induced differences in droplet kinetic energy and mass distribution: higher curvature promotes spreading along the *x*-direction, where more momentum is transmitted, while spreading along the *y*-direction is constrained, enhancing asymmetry.

However, Fig. 6c indicates that this curvature-induced asymmetry mainly affects the spatial extent of spreading and has little effect on the time to reach maximum spread. Specifically, Δ*t* changes little with C^*^, but increases substantially with *We*; as *We* rises from 49 to 168, Δ*t* increases by 89.21%. This demonstrates that Δ*t* is primarily governed by inertial effects: higher *We* increases droplet impact velocity and inertia, and combined with the *x*-direction’s inherent momentum advantage, this further amplifies spreading in *x*, significantly affecting Δ*t*.

Figure 6d shows that *δ* is largely insensitive to *We* and C^*^, being mainly influenced by leaf surface microstructure or wettability. Even across three repeats, the final stabilized D^*^ varies. Together with observations in Fig. 5, these results indicate that after impacting a curved surface, droplet dynamics are controlled by the interplay of inertia, surface tension, and surface structure. At high *We*, inertia dominates, determining the maximum spread shape, while during retraction, surface tension governs. Local pinning from microstructural features can impede retraction and, if sufficiently strong, cause film rupture and satellite droplet formation, resulting in irregular final shapes.

#### Effects of C^*^ and *We* on droplet spreading behavior

**Fig. 7:**
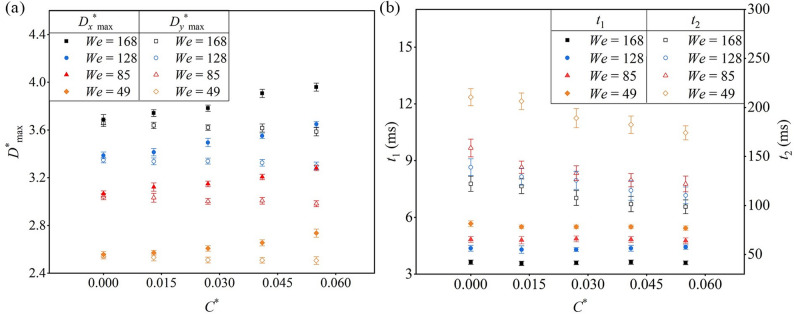
Droplet spreading behavior varies with C^*^ and *We*: (**a**) D^*^_max_; (**b**) contact time

Droplets exhibit regular behavior at maximum spreading, which is further analyzed here. Figure 7a shows the variation of maximum spreading diameters under different *We* and C^*^. Both *D*_x_^*^_max_ and *D*_y_^*^_max_ increase with *We*; for C^*^ = 0, raising *We* from 49 to 168 enlarges both by ~ 30%, consistent with the theoretical prediction that inertial forces dominate and grow with *We*.

On the other hand, increasing leaf curvature introduces anisotropy: at *We* = 168, when C^*^ = 0.055 compared with the flat surface, increases *D*_x_^*^_max_ by 6.89% while slightly reducing *D*_y_^*^_max_ by 1.95%. Theoretically, curvature influences spreading via two mechanisms: (1) the curvature correction function Г(C^*^) decreases with C^*^, reducing the effective tangential surface tension along *y* and limiting *D*_y_^*^_max_; (2) the asymmetry factor *λ* rises with C^*^, redistributing more kinetic energy along *x*, enhancing *D*_x_^*^_max_. The observed “*x*-direction enhancement and *y*-direction reduction” directly reflects these curvature-induced effects on local contact line forces.

Furthermore, high *We* also intensifies inertial effects, amplifying thin-film deformation and edge instability. Experiments on static leaves indicate splashing at *We* ≈ 168, which is taken as the critical *We* under the given surface conditions and serves as a reference for subsequent vibrating-leaf experiments.

The contact time between droplets and the leaf surface is critical for pesticide adhesion. The spreading time *t*_1_ is defined as the moment when the droplet reaches its maximum *x*-direction diameter, and the retraction time *t*_2_ corresponds to its return to a stable state. As shown in Fig. 7b, *t*_1_ is only weakly influenced by C^*^, whereas *t*_2_ decreases notably with increasing curvature. At *We* = 168, C^*^ = 0.055 reduces *t*_2_ by 0.92% relative to a flat surface. This agrees with the theoretical framework: Eq. (8) indicates that spreading is inertia-dominated, and curvature primarily redistributes kinetic energy without altering overall inertial dissipation, resulting in minimal change in *t*_1_. Retraction, however, is governed by surface tension, and larger curvature enhances dissipative pathways, thereby shortening *t*_2_. This suggests that for leaves with C^*^ ≥ 0.055, increasing liquid viscosity may help prolong *t*_2_ and reduce coalescence losses.

Additionally, both *t*_1_ and *t*_2_ decline with increasing *We*. For C^*^ = 0, raising *We* from 49 to 168 shortens *t*_1_ and *t*_2_ by 35.88% and 41.96%, respectively. Higher *We* provides greater initial kinetic energy, accelerating spreading and compressing the liquid film, which strengthens momentum transfer and outward flow. Thus, high *We* facilitates rapid expansion and larger coverage but may reduce contact time to a level insufficient for effective interaction with leaf microstructures.

**Fig. 8:**
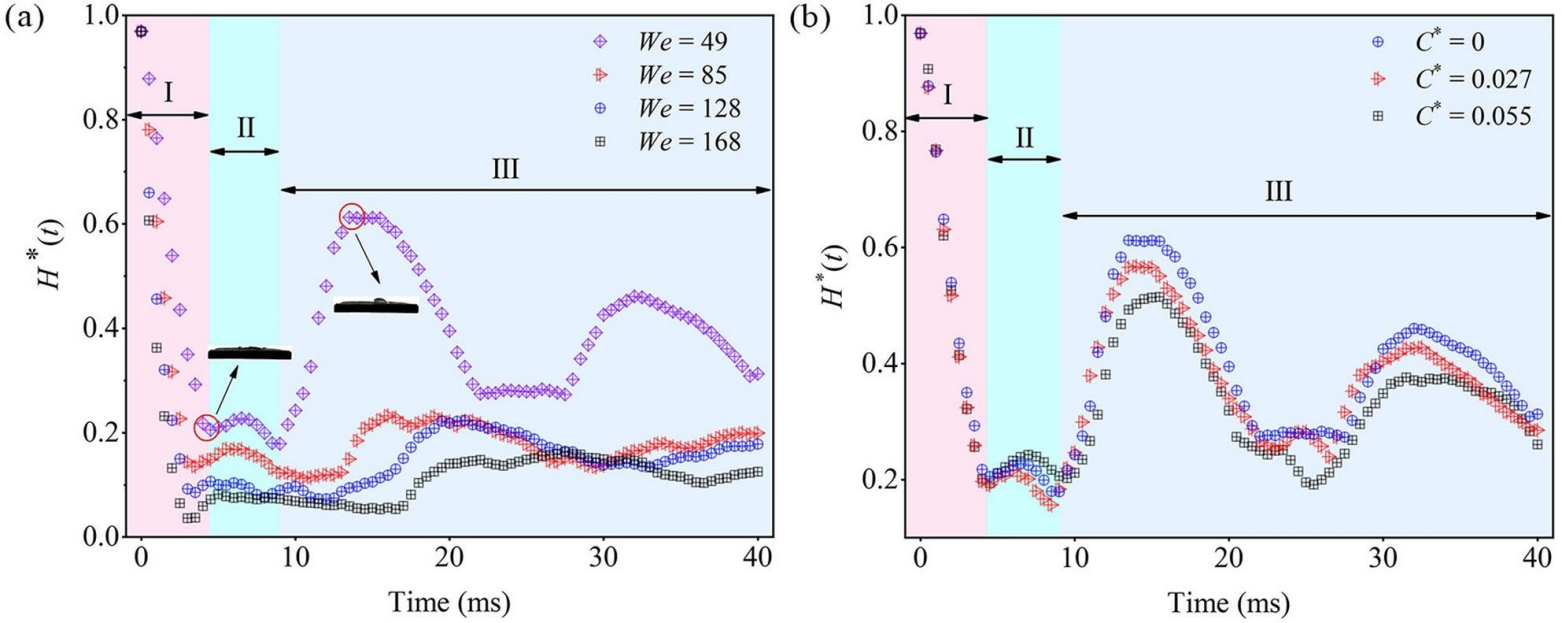
Time-dependent curve of H^*^: (**a**) influence of *We*; (**b**) influence of C^*^

Static leaf surface experiments revealed that variations in *t*_2_ with *We* and C^*^ are primarily driven by droplet oscillations. Since droplet height (*H*) can indirectly reflect its dynamic evolution, the dimensionless height H^*^ = *H* / *D*_0_ was used to eliminate droplet size effects and analyze temporal variation (Fig. 8a). Using *We* = 49 as an example, the H^*^(*t*) curve can be divided into three stages:

Stage I: Immediately after impact, H^*^ rapidly decreases to its minimum value H^*^_min_, corresponding to maximum spreading. H^*^_min_ increases with *We*, consistent with the trend of D^*^_max_.

Stage II: Following maximum spreading, H^*^ exhibits oscillations. Higher *We* prolongs these oscillations due to greater kinetic energy opposing surface tension.

Stage III: H^*^ rebounds to its maximum value H^*^_max_ during retraction. H^*^_max_ decreases as We increases, with pronounced differences observed between *We* = 49 and 85, indicating greater instability at low *We*. Thereafter, H^*^ undergoes periodic fluctuations before reaching stabilization.

Overall, low *We* droplets show stronger oscillations and longer *t*_2_, while high *We* droplets exhibit weaker oscillations and shorter *t*_2_.

To examine the effect of C^*^ on H^*^, the case of *We* = 49 with pronounced H^*^ fluctuations was analyzed (Fig. 8b). C^*^ shows little influence during Stages I and II, consistent with its minimal effect on *t*_1_. In Stage III, however, H^*^_max_ decreases notably with increasing C^*^. For example, at C^*^ = 0.055, H^*^_max_ is 15.93% lower than at C^*^ = 0. This reduction arises because higher C^*^ decreases the equivalent contact angle, enhances surface energy effects, and enlarges the droplet–leaf contact area, thereby increasing adhesion dissipation and reducing H^*^_max_.

### Kinetic behavior of the droplet on vibrating leaf surfaces

**Fig. 9:**
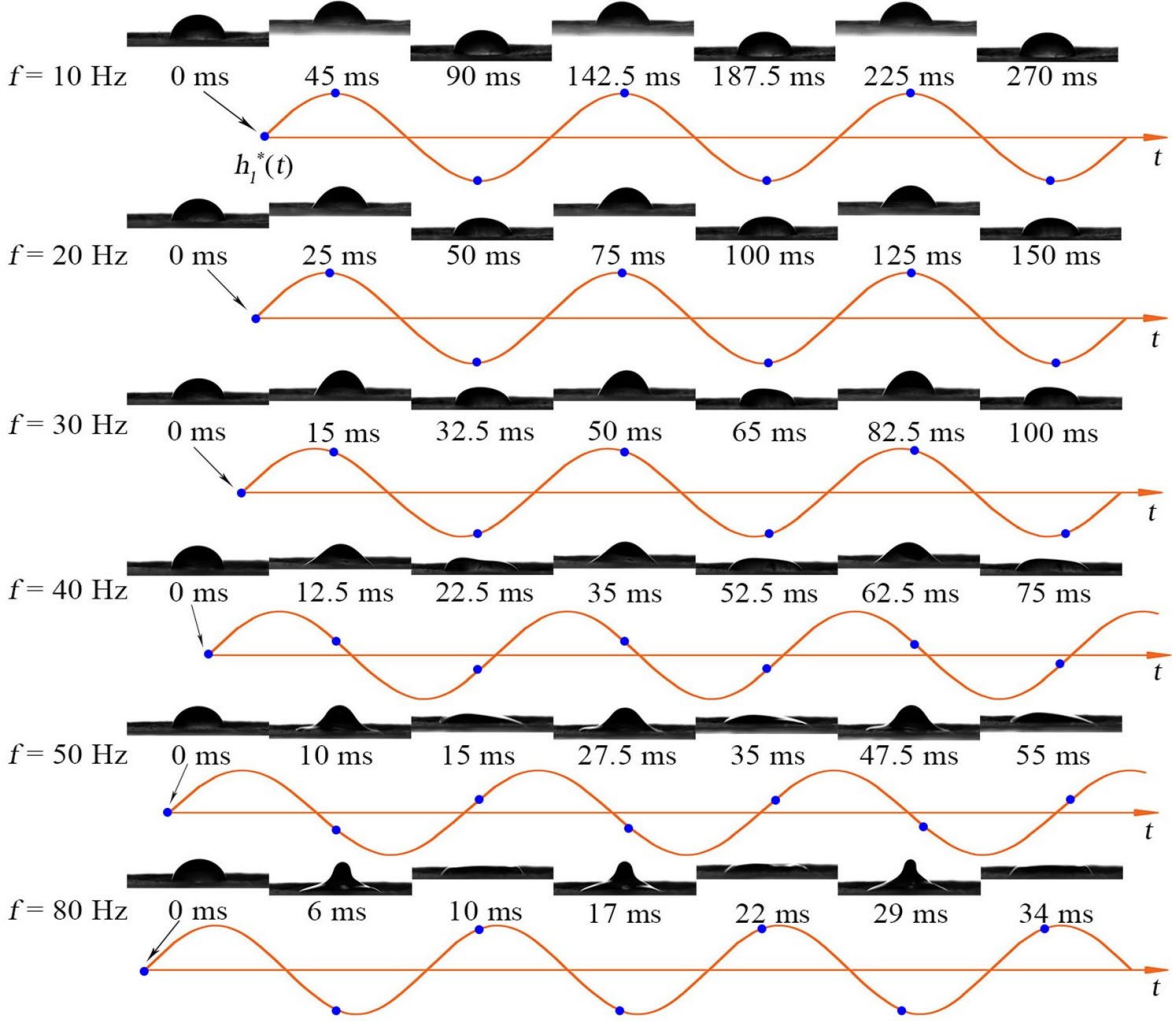
Kinetic behavior of droplets at different *f* (Droplet images represent the initial shape, maximum elongation, and maximum compression of droplet height during oscillation. Curves represent the leaf surface displacement. Blue dots represent the corresponding time points of the droplet images.)

To investigate the effect of leaf vibration on droplet dynamics, experiments were conducted across a range of vibration frequencies *f*, with droplet deformation and motion recorded by high-speed imaging. For capsicum leaves, measurements showed *f* < 30 Hz at wind speeds ≤ 9 m/s, consistent with previous studies [19, 20, 27–29] reporting *f* ≤ 30 Hz under low wind conditions (≤ 10 m/s). To also account for higher wind speeds and different crop species, the frequency range was extended to *f* ≤ 80 Hz. As shown in Fig. 9, droplet images represent the initial morphology and maximum extension/compression, while curves show leaf surface displacement. Blue dots mark droplet images. Results indicate that with increasing *f* from 10 to 80 Hz, droplet displacement peaks progressively lag behind the leaf surface, revealing a transition from co-directional motion at low frequencies to counter-directional motion at high frequencies.

#### Effect of vibration frequency *f* on droplet spreading

**Fig. 10:**
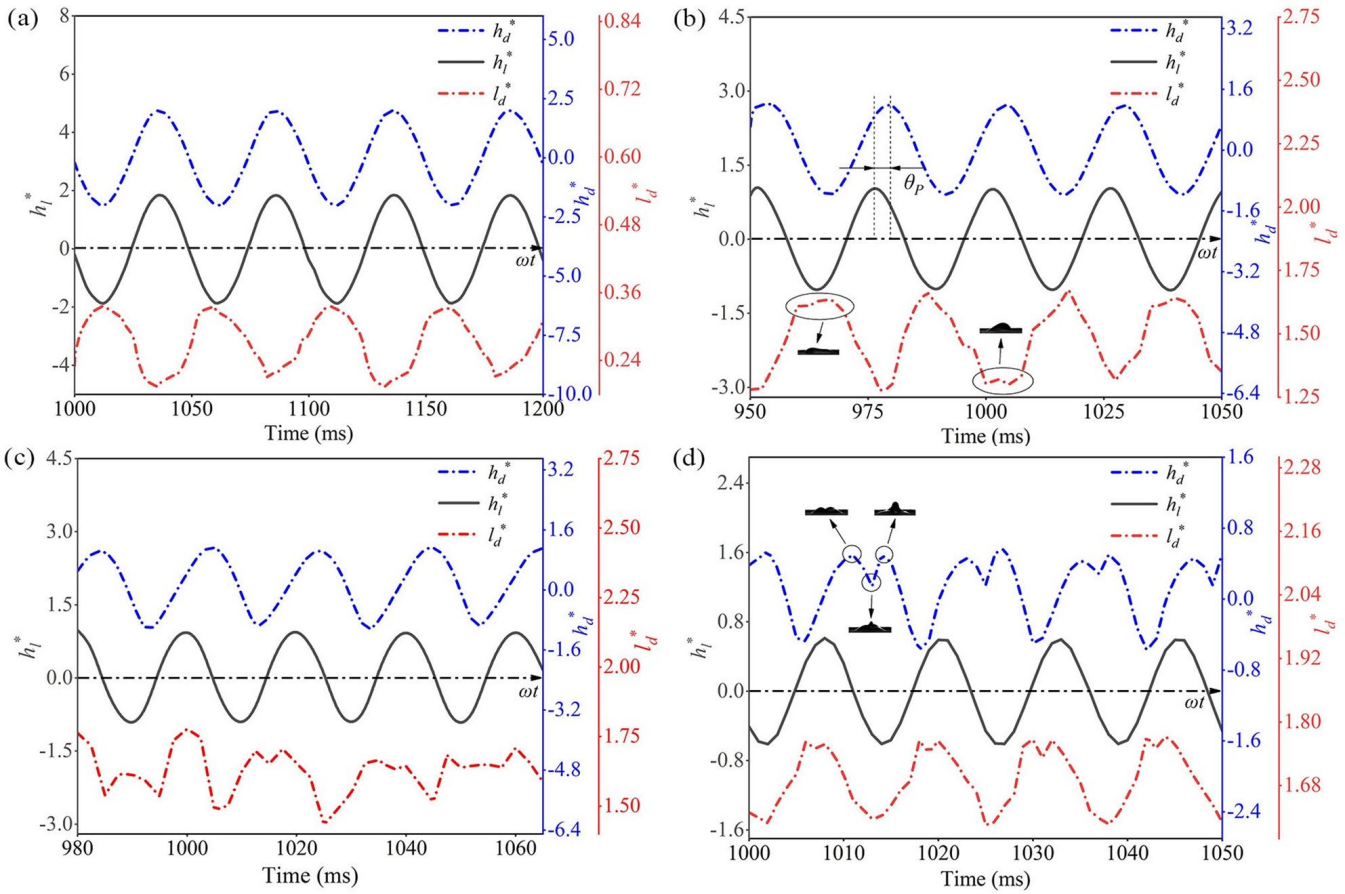
Temporal variation of *h*_l_^*^(*t*), *h*_d_^*^(*t*) and *l*_d_^*^(*t*) at different *f*: (**a**) *f* = 20 Hz; (**b**) *f* = 40 Hz; (**c**) *f* = 50 Hz; (**d**) *f* = 80 Hz

To further examine the effect of leaf surface vibration on droplet dynamics, Fig. 10 presents the variations of *h*_l_^*^(*t*), *h*_d_^*^(*t*) and *l*_d_^*^(*t*) at different frequencies. As *f* increases from 20 to 80 Hz, *h*_d_^*^(*t*) and *l*_d_^*^(*t*) become increasingly irregular, with *l*_d_^*^(*t*) showing the most pronounced fluctuations. At 20 Hz, both curves are relatively smooth and close to sine waves. By 40 Hz, *l*_d_^*^(*t*) develops distinct peaks and troughs, consistent with lateral oscillations near resonance, where the maximum lateral displacement Δ*l*_d_^*^(*t*)_max_ is about 0.38. This agrees with Rohde et al. [30], who reported that vertically vibrated droplets exhibit lateral motion due to displacement of the center of mass, while contact point motion remains limited. At 80 Hz, both *h*_d_^*^(*t*) and *l*_d_^*^(*t*) display irregular double peaks (Fig. 10d). This indicates that at high frequencies, droplet response lags behind leaf vibration, breaking synchronization and inducing strong internal shear and inertial imbalance. Here, inertial forces dominate over surface tension and viscosity, and vibration energy transfers into surface waves, forming dual peaks that gradually merge into a single peak under surface tension before continuing to grow.

**Fig. 11:**
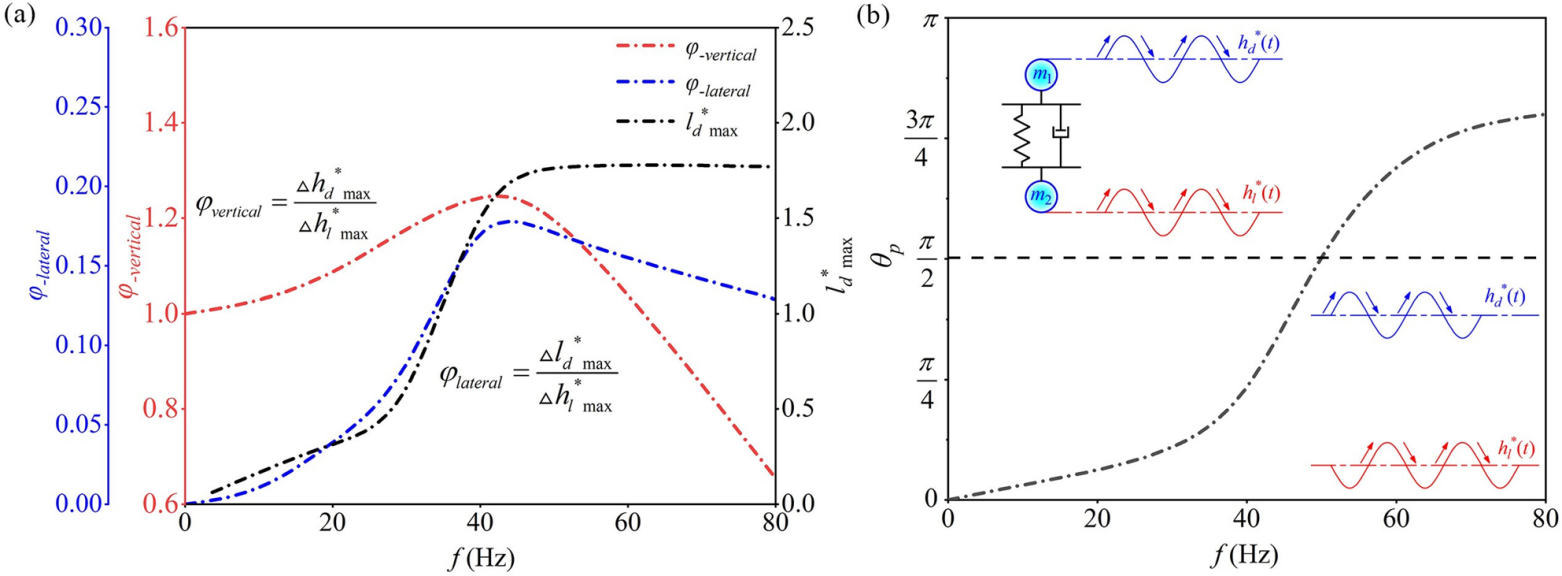
Variation of droplet parameters with *f*: (**a**) amplitude amplification factor; (**b**) phase angle

Figures 9 and 10 show clear variations in Δ*h*_d_^*^(*t*)_max_, Δ*h*_l_^*^(*t*)_max_ and Δ*l*_d_^*^(*t*)_max_ at different frequencies *f*, reflecting the stretching and compression of droplets induced by leaf surface vibrations. To quantitatively assess this effect, the ratio of Δ*h*_d_^*^(*t*)_max_ to Δ*h*_l_^*^(*t*)_max_ is defined as the vertical amplitude amplification coefficient (*φ*_−vertical_), while the ratio of Δ*l*_d_^*^(*t*)_max_ to Δ*h*_l_^*^(*t*)_max_ is defined as the lateral amplitude amplification coefficient (*φ*_−lateral_). Given that previous studies [18–20] consistently reported that surface vibrations promote droplet spreading, this study focused on *l*_d_^*^(*t*)_max_ and Δ*l*_d_^*^(*t*)_max_ to characterize the enhancement of droplet spreading under leaf surface vibrations. The corresponding computational results are shown in Fig. 11a.

As shown in Fig. 11a, when *f* < 40 Hz, *φ*_−vertical_ ≈ 1 and *φ*_−lateral_ ≈ 0, indicating nearly synchronous droplet-leaf motion (Fig. 10a). In this regime, droplet deformation is minimal and energy transfer remains weak, favoring smooth spreading and deposition with reduced splashing. When 40–50 Hz, both *φ*_−vertical_ and *φ*_−lateral_ reach their maxima (1.257 and 0.194), revealing a resonance state. Strong droplet deformation and highly efficient energy transfer promote rapid spreading and larger coverage, although additives are needed to suppress breakup and satellite droplets. At *f* > 50 Hz, *φ*_−vertical_ approaches 0 while *φ*_−lateral_ approaches 1, implying opposite-phase motion and excessive deformation that may lead to droplet detachment (Fig. 10d). Therefore, leaf vibration frequencies above 40 Hz should be avoided in practical spraying to reduce splashing and pesticide loss. Additionally, *l*_d_^*^(*t*)_max_ increases with f and peaks at 1.7 within the resonance range (40–50 Hz) before stabilizing, indicating that low-frequency vibrations have limited wetting enhancement, whereas high-frequency vibrations markedly strengthen droplet spreading.

In a series of preliminary experiments on vibrating leaves, it was observed that as *f* increased from 10 Hz to 80 Hz, the *h*_d_^*^(*t*) and *h*_l_^*^(*t*) curves gradually shifted from being in-phase to out-of-phase, reflecting a phase lag between the droplet and leaf motion. To quantify this lag, the phase difference *θ*_*p*_ was introduced (Fig. 10b) and calculated from the peak time difference within each vibration cycle. The results (Fig. 11b) show that when *f* < 40 Hz, *θ*_*p*_ approaches 0, indicating synchronous motion of the droplet and leaf; for 40 Hz ≤ *f* ≤ 50 Hz, *θ*_*p*_ approaches *π*/2, indicating the onset of counter-phase motion; and for *f* > 50 Hz, *θ*_*p*_ approaches *π*, indicating fully out-of-phase motion. The frequency corresponding to *θ*_*p*_ ≈ *π*/2 (*f* ≈ 45 Hz) can thus be approximately regarded as the system’s resonance frequency.

To further quantify the droplet-leaf system dynamics, frequencies near the natural frequency were selected as characteristic points. At *f* = 60 Hz, the frequency ratio *λ* ≈ 1.333 and the phase difference *θ*_*p*_ ≈ 3*π*/4. Substituting these values into Eq. (15) yields an equivalent damping ratio *ξ* ≈ 0.136. Subsequently, the amplitude at *f* = 30 Hz (*φ*_*vertical*_ ≈ 1) was used for verification: with *λ* ≈ 0.667 and *ξ* ≈ 0.14 substituted into Eq. (14), the calculated result agreed with the experimental measurement. By combining theoretical predictions and experimental observations, the system damping ratio was determined to be *ξ* ≈ 0.14. This indicates that the system is lightly damped, with energy dissipation primarily arising from internal droplet viscosity and contact line hysteresis. The observed phase lag not only reflects the dynamic energy transfer between the droplet and leaf but can also be interpreted using a forced-damped model. The quantified damping ratio provides a basis for assessing droplet detachment risk at different vibration frequencies.

#### Combined effects of *We*, C^*^, and *f* on droplet impact dynamics

This section examines the combined effects of *We*, C^*^, and *f* on droplet impact and vibration behavior on the leaf surface, extending the preceding analysis of droplet dynamics. Using Design Expert 8.0.6, quadratic regression was applied to the experimental results of droplet spreading. Based on this, quadratic polynomial response surface model for the maximum spreading factor (D^*^_max_) was established, as presented in Eq. (16).

16 $$\begin{aligned} {D^*}_{{\hbox{max} }}&= - 15.832+0.084 \cdot We - 7.651 \cdot {C^*}\\&+0.561 \cdot f - 8.929 \times {10^{ - 3}} \cdot We \cdot {C^*}\\&- 3.750 \times {10^{ - 4}} \cdot We \cdot f \\& +0.107 \cdot {C^*} \cdot f \\&- 1.744 \times {10^{ - 4}} \cdot W{e^2}+103.316 \cdot {C^*}^{2} \\&- 5.590 \times {10^{ - 3}} \cdot {f^2} \end{aligned}$$

**Fig. 12:**
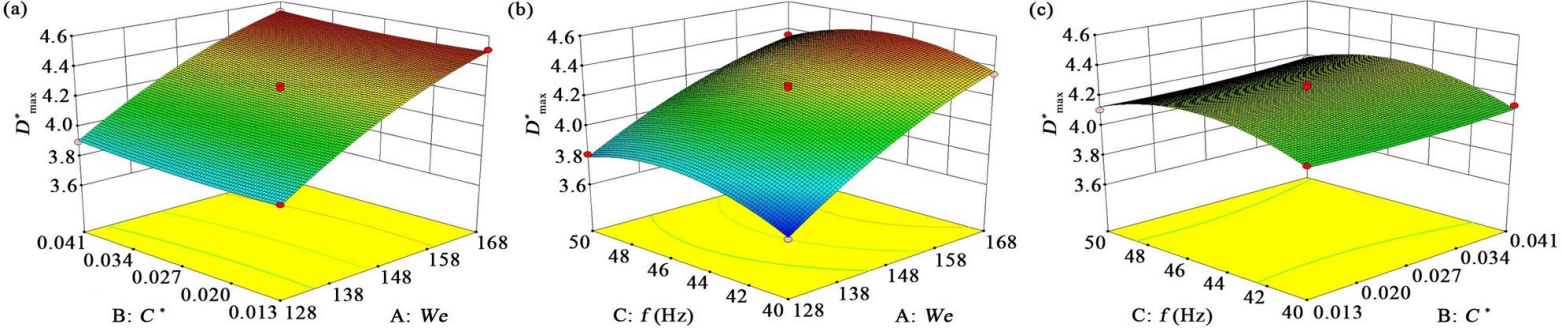
Response surface plots showing the multifactor interactions influencing D^*^_max_: (**a**) Interaction between *We* and C^*^ on D^*^_max_ at *f* = 45 Hz; (**b**) Interaction between *We* and *f* on D^*^_max_ at C^*^ = 0.027; (**c**) Interaction between C^*^ and *f* on D^*^_max_ at *We* = 148

Figure 12 illustrates the interactive effects of *We*, C^*^, and *f* on D^*^_max_. As shown in Fig. 12a, when *f* is fixed, D^*^_max_ increases notably with rising *We*. For example, at C^*^ = 0.013, D^*^_max_ grows from 3.65 to 4.53 as *We* increases from 128 to 168. In contrast, at *We* = 128, raising C^*^ from 0.013 to 0.041 causes only a slight increase in D^*^_max_ from 3.87 to 3.90. This demonstrates that *We* is the dominant factor governing D^*^_max_, as it reflects the competition between inertial and surface tension forces. At high *We*, inertial effects dominate, increasing droplet kinetic energy and enhancing spreading, while at low *We*, surface tension suppresses spreading.

Figure 12b shows that when C^*^ is fixed, D^*^_max_ first increases and then decreases with *f*. At *f* = 40 Hz, the phase difference *θ*_*p*_ is small, indicating near-synchronous motion between the droplet and the leaf, resulting in limited spreading enhancement. At *f* = 45 Hz (resonance frequency), D^*^_max_ reaches approximately 3.86, and the vibration amplitude amplification peaks, providing the droplet with extra kinetic energy that greatly promotes spreading. Beyond the resonance frequency, the growing phase lag reduces energy transfer efficiency, thereby weakening spreading.

Overall, D^*^_max_ shows a positive correlation with C^*^, as greater curvature tends to constrain spreading. However, under vibration conditions near the droplet’s natural frequency, vibration-induced kinetic energy dominates liquid film motion, reducing the geometric influence of C^*^. As shown in Fig. 12c, the impact of *f* on D^*^_max_ is much stronger than that of C^*^, indicating that leaf vibration characteristics play a key role in determining droplet spreading behavior.

During the dynamic leaf surface experiments, droplet splashing was observed even at *We* values below 168, the critical threshold determined for static leaves, indicating that leaf vibration lowers the splashing onset condition. To quantify the influence of *f* on splashing, the response surface model based on the Box-Behnken design was used to extract the critical *We* at different *f* values. The results show that when *f* = 40, 45, and 50 Hz, the critical *We* values are approximately 152, 132, and 144, respectively, about 9.5%, 21.4%, and 14.3% lower than that of the static surface (*We* ≈ 168).

This demonstrates that leaf vibration promotes splashing, with the strongest effect near the resonance frequency where the critical *We* is minimized. The mechanism arises from the coupling between leaf vibration and droplet impact energy: vibration provides additional kinetic energy to the droplet, leading to excessive stretching and the formation of a thinner, elongated liquid film. At resonance, energy transfer efficiency peaks, greatly increasing droplet kinetic energy and disrupting surface tension balance. This results in waveform instabilities along the contact line, which intensify at higher *We* and ultimately trigger droplet breakup and splashing.

The reliability of the regression model was assessed using model adequacy indicators. As shown in Table 3, the D^*^_max_ model achieved an *R*^2^ value of 0.9959, explaining 99.59% of the variation in the maximum droplet spreading diameter. Even after adjustment for degrees of freedom, the model maintained an excellent goodness of fit. The coefficient of variation (C.V. = 0.56%) was far below the 5% threshold, indicating high experimental repeatability and minimal random error. The adequate precision value of 48.955 greatly exceeded the acceptable limit of 4, demonstrating a strong signal-to-noise ratio and reliable predictive capability.

**Table 3:** Analysis of variance (ANOVA) for the regression model

Variance sources	Sum of squares	Df	Mean square	F values	*P* values
Model	0.92	9	0.10	188.60	< 0.000 1
*A*–*We*	0.79	1	0.79	1470.00	< 0.000 1
*B*–C^*^	3.20 × 10^–3^	1	3.20 × 10^–3^	5.93	0.045 1
*C*–*f*	6.05 × 10^–3^	1	6.05 × 10^–3^	11.20	0.012 3
*AB*	2.50 × 10^–5^	1	2.50 × 10^–5^	0.046	0.835 8
*AC*	5.63 × 10^–3^	1	5.63 × 10^–3^	10.42	0.014 5
*BC*	2.25 × 10^–4^	1	2.25 × 10^–4^	0.42	0.539 2
*A* ^2^	0.02	1	0.020	37.93	0.000 5
*B* ^2^	1.73 × 10^–3^	1	1.73 × 10^–3^	3.20	0.116 9
*C* ^2^	0.08	1	0.08	152.28	< 0.000 1
Residual	3.78 × 10^–3^	7	5.40 × 10^–4^		
Lack of fit	2.30 × 10^–3^	3	7.67 × 10^–4^	2.07	0.246 6
Pure error	1.48 × 10^–3^	4	3.70 × 10^–4^		
Total sum	0.92	16			
Model adequacy indicators	*R* ^2^	Adjusted *R*^2^	Predicted *R*^2^	C.V.	Adequate Precision
	0.995 9	0.990 6	0.957 5	0.56%	48.955

Moreover, the *P*-value results confirmed that *We*, C^*^, and *f* all exert significant effects on D^*^_max_ (*P* < 0.05), with the order of influence being *We* > *f* > C^*^. Overall, *We* was identified as the dominant factor, followed by *f*. Therefore, in practical field spraying, *We* should be prioritized for control, for instance, by reducing droplet impact velocity or diameter to maintain *We* < 132, and vibration frequencies near the resonance range should be avoided (preferably keeping *f* < 40 Hz).

In summary, *We* is the primary control parameter, followed by *f*. For field spraying, *We* should be maintained below 132. According to Eq. (1), this requires controlling droplet impact velocity to 3–5 m/s under typical conditions (*D*_0_ = 150–300 μm, *σ* = 0.03 N/m), and further reducing it to 2–3 m/s for larger droplets (> 500 μm). Thus, lowering nozzle pressure or reducing operational airflow provides an effective means to suppress high *We* splashing by directly decreasing impact velocity.

Because surfactants reduce surface tension and increase *We*, spray pressure should be further lowered or smaller droplets selected when adjuvants are used. To prevent leaf vibration from entering the resonance regime, the excitation frequency should be kept below 40 Hz. In field applications, leaf excitation is mainly induced by the spray airflow; therefore, controlling airflow velocity can effectively limit leaf vibration. For example, maintaining an air-assisted spray velocity of 6–10 m/s can reduce the aerodynamic response frequency of leaves and avoid high-frequency vibrations that hinder deposition.

Overall, the theoretical critical limits (*We* < 132 and *f* < 40 Hz) can be directly regulated through nozzle pressure, droplet size selection, and airflow optimization, offering clear operational guidance for improving deposition efficiency in field spraying.

#### Validation of the predictive capability of regression model

To further validate the practical applicability of the established D^*^_max_ regression model and ensure its reliability in predicting new, unfitted operating conditions, independent validation experiments were conducted following the same experimental procedures as those used for model development. Based on the factor level ranges defined in the original Box-Behnken design (*We* = 128–168, C^*^= 0.013–0.041, and *f* = 40–50 Hz), five validation points were selected to represent low, medium, and high levels of each factor. The experimentally measured values were compared with the model predictions, and the prediction performance was quantitatively evaluated using absolute and relative errors as assessment metrics. The results are summarized in Table 4.

**Table 4:** Validation experiment plan and results

Number	We	C^*^	f	Predictive value of D^*^_max_	Measured value of D^*^_max_	Absolute error	Relative error
1	125	0.013	35	3.04	3.57	0.53	14.85%
2	137	0.041	40	3.83	3.91	0.08	2.05%
3	174	0.027	55	3.85	4.44	0.59	13.29%
4	148	0.013	45	4.17	4.23	0.06	1.42%
5	162	0.041	50	4.26	4.32	0.06	1.39%

The results show that the D^*^_max_ model demonstrates high predictive accuracy within the original fitted factor range. At validation points 2, 4, and 5, the predicted values closely matched the experimental results, with relative errors of 2.05%, 1.42%, and 1.39%, all below 5%. This confirms that the model effectively captures the coupled effects of *We*, C^*^, and *f* on D^*^_max_ within this range. However, when factor levels exceeded the fitted range (e.g., validation points 1 and 3), the relative errors increased to 14.85% and 13.29%, indicating reduced accuracy under extrapolation. This limitation arises because the Box-Behnken design, based on a quadratic polynomial form, is suitable for interpolation within the design space but cannot fully represent the nonlinear dynamics of the droplet-leaf system beyond it. Overall, the model provides reliable predictions within the designed range and can be used to analyze and optimize droplet spreading behavior. For broader applicability, future work could incorporate boundary-point experiments or higher-order nonlinear terms to improve model robustness.

## Conclusions

Static-leaf experiments show that leaf curvature (C^*^) induces anisotropic droplet spreading, with *D*_x_^*^_max_ increasing by 6.89% and *D*_y_^*^_max_ decreasing by 1.95%. Higher *We* (*We* ≥ 168) reduce the spreading time by about 35.9% but limit droplet contact and retention on the leaf surface. For high-curvature leaves (C^*^ ≥ 0.055), increasing spray-liquid viscosity helps reduce losses caused by retraction and coalescence. A critical *We* of approximately 168 is identified for splashing on static leaves, providing a reference for optimizing spraying parameters.

Vibrating leaf surface experiments show that droplet dynamics are highly dependent on leaf vibration frequency. When *f* < 40 Hz, the phase difference *θ*_*p*_ is near zero and droplets move almost in phase with the leaf, remaining stably deposited. Within the resonance range of 40 to 50 Hz, *θ*_*p*_ approaches *π*/2, droplet deformation, amplitude, and spreading reach their maxima, and the risk of splashing increases. When *f* > 50 Hz, *θ*_*p*_ approaches *π*, enhancing wetting but increasing the likelihood of droplet detachment. The estimated damping ratio of approximately 0.14 indicates a lightly damped droplet-leaf system, providing a quantitative basis for assessing detachment risk under different vibration conditions.

A predictive model for the maximum spreading diameter (D^*^_max_) under combined effects of *We*, C^*^, and *f* shows that all factors are significant (*P* < 0.05), with their influence ranked as *We* > *f* > C^*^. The critical *We* at *f* = 40, 45, and 50 Hz is reduced by 9.5%, 21.4%, and 14.3% compared with static leaves, confirming that leaf vibration, especially near resonance, promotes droplet splashing.

For field applications, maintaining *We* below 132 is essential; under typical spraying conditions, this corresponds to maintaining the droplet impact velocity within 3–5 m/s, while larger droplets (greater than 500 μm) require a further reduction in impact velocity to 2–3 m/s. To avoid resonance (*f* < 40 Hz), the airflow in air-assisted spraying should be kept within 6–10 m/s. These thresholds, *We* < 132 and *f* < 40 Hz, can be directly regulated through adjustments of nozzle pressure, droplet size, and airflow, providing practical guidance for improving deposition efficiency in field spraying.

## Data Availability

The datasets used and/or analyzed during the current study are available from the corresponding authors upon reasonable request.
